# Transvaginal Hybrid NOTES Procedure for Treatment of Gallstone Ileus

**DOI:** 10.1155/2016/9513874

**Published:** 2016-06-26

**Authors:** Takuya Shiraishi, Naoki Tomizawa, Tatsumasa Andoh, Kazuhisa Arakawa, Yasuaki Enokida, Naoya Ozawa

**Affiliations:** Department of Surgery, Japanese Red Cross Maebashi Hospital, 3-21-36 Asahi-cho, Maebashi, Gunma 371-0014, Japan

## Abstract

Gallstone ileus is a rare mechanical bowel obstruction, and previously reported cases have been treated laparoscopically with good results. Although transvaginal hybrid NOTES without a minilaparotomy has been reported to decrease the incidence of surgical wound complications, to our knowledge, this procedure has not been used previously to treat gallstone ileus. We present a case of a 63-year-old woman who underwent transvaginal hybrid NOTES procedure for treatment of gallstone ileus. This case was admitted to our hospital following acute-onset abdominal pain and vomiting. We diagnosed gallstone ileus with cholecystoduodenal fistula by computed tomography and performed totally laparoscopic surgery using only three 5 mm abdominal ports with transvaginal specimen extraction and enterectomy. The patient's postoperative course was uneventful, and laparoscopic cholecystectomy and fistula repair were performed 8 months after the initial surgery. The patient experienced additional pain relief and good cosmetic outcomes. In conclusion, using transvaginal hybrid NOTES may become a future option to minimize the invasiveness of other laparoscopic procedures.

## 1. Introduction

Gallstone ileus is a rare bowel obstruction accounting for 1%–3% of all cases of mechanical obstruction of the small bowel [1]. Gallstone ileus is an abdominal emergency, usually treated surgically. Open enterolithotomy has been the surgery of choice, but many cases are now operated laparoscopically with good results [2, 3]. However, conventional laparoscopic surgery for gallstone ileus requires an abdominal minilaparotomy for gallstone extraction. Recently, transvaginal hybrid natural orifice transluminal endoscopic surgery (NOTES), which does not involve a minilaparotomy, has been associated with decreased surgical wound complications [4, 5]. We present a case of transvaginal hybrid NOTES procedure for treatment of gallstone ileus followed by laparoscopic cholecystectomy and fistula repair, 8 months after the initial operation.

## 2. Case Presentation

**Figure 1:**
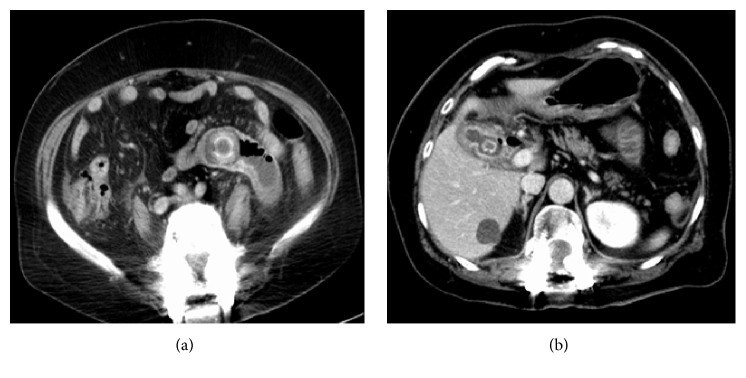
Computed tomography (CT) images showing (a) small bowel obstruction by a partially calcified object in the jejunum and (b) inflammation around the gallbladder, with a gallstone, and thickening between the gallbladder and the duodenal walls. Air-fluid levels in the gallbladder were also seen.

A 63-year-old woman with mechanical bowel obstruction was referred to our emergency department. She complained of abdominal pain and vomiting that began that morning, and intermittent abdominal pain in the preceding 2 weeks. There was no history of gallbladder disease. On admission, her body mass index was 24 kg/m^2^, she did not have a fever, and her abdomen was distended without signs of peritonitis. Laboratory data indicated an elevated white blood cell count of 34.4 × 10^9^/L (range, 4.0–9.0 × 10^9^/L), and C-reactive protein level of 19.24 nmol/L (range, 0.00–2.86 nmol/L). Computed tomography (CT) revealed small bowel obstruction by a partially calcified object in the jejunum (Figure 1(a)). Air-fluid levels in the gallbladder were also detected on CT (Figure 1(b)). A cholecystoduodenal fistula resulting from chronic cholelithiasis and gallstone ileus were suspected.

She underwent conservative therapy with antibiotics, and a nasogastric tube was inserted for bowel decompression; however, her abdominal pain did not improve, despite the use of analgesics. Repeat CT revealed that the bowel dilation had improved slightly but that the gallstone position was unchanged.

The patient and her family consented to the recommended operation and also signed a detailed consent form. They also understood the potential risks of the transvaginal approach, and consent was obtained to publish the details of the patient's case.

**Figure 2:**
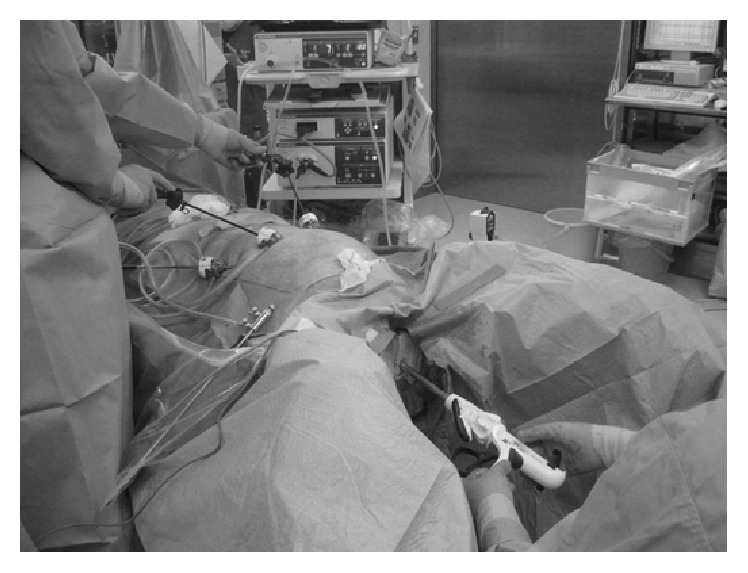
Intraoperative image showing the liner stapler being inserted into the abdominal cavity via the vaginal 12 mm port inserted into the cap.

**Figure 3:**
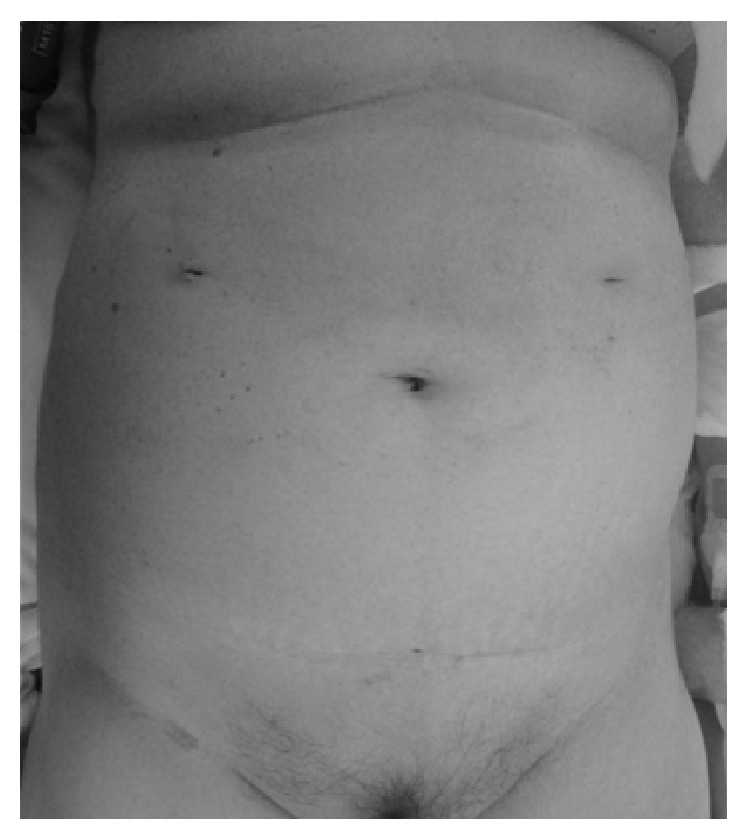
Photograph showing the postoperative scars for the three 5 mm ports that were inserted into the abdomen.

**Figure 4:**
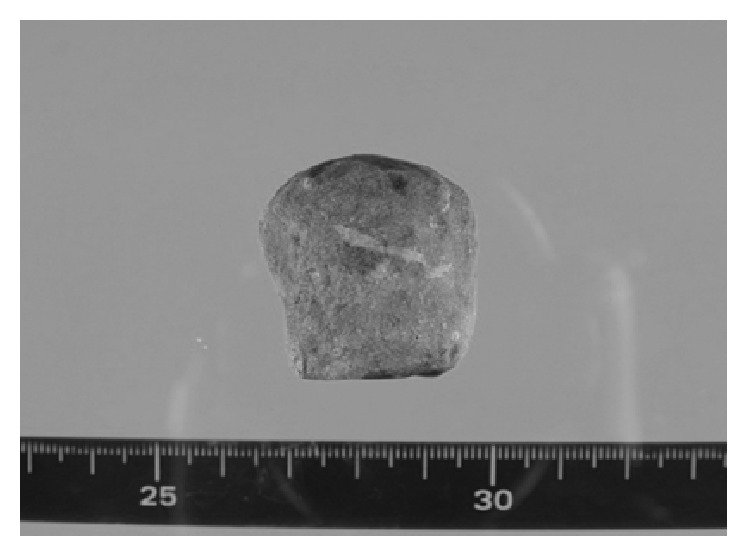
The extracted gallstone measuring 3.5 × 3.0 × 3.0 cm.

After induction of general anesthesia, the vagina was washed with 500 mL of 0.02% benzalkonium chloride solution. A 5 mm port was inserted at the navel visually, and pneumoperitoneum was established. Two 5 mm ports for the laparoscopic instruments were inserted into the upper abdomen, and laparoscopic exploration confirmed the bowel obstruction approximately 70 cm from the ligament of Treitz. The bowel was thinning at the obstruction, and we were concerned about perforation, postoperatively; therefore, we decided to perform enterectomy with gallstone extraction. After placing the patient in the lithotomy position, a posterior colpotomy was performed using an ultrasonic scalpel under transvaginal guidance. An Alexis bundle wound protector with cap (Alexis Laparoscopic System with Kii Fios First Entry®; Applied Medical, Rancho Santa Margarita, CA, USA) was carefully inserted into the abdominal cavity, transvaginally, through the posterior colpotomy. The white ring of the system was covered with the cap that was inserted into a 12 mm port, maintaining pneumoperitoneum. A liner stapler was then inserted into the abdominal cavity via the vaginal port, and the proximal and distal jejunum at the site of the obstruction were transected (Figure 2). Next, a grasping forceps was inserted into the abdominal cavity, transvaginally, to grasp and remove the specimen with the gallstone. A liner stapler was again inserted into the abdominal cavity, and functional end-to-end anastomosis was performed. The posterior colpotomy incision was closed transvaginally. The laparoscopic surgery for gallstone ileus was completed using three 5 mm abdominal ports (Figure 3), and the size of the extracted gallstone was 3.5 × 3.0 × 3.0 cm (Figure 4). The surgical duration was 162 min, with minimal blood loss. The patient was allowed to drink fluids on postoperative day 2, and no analgesics were administrated after postoperative day 2. She recovered uneventfully and was discharged on postoperative day 11. Eight months postoperatively, although she had experienced no recurrence of gallstone ileus and cholecystitis, laparoscopic cholecystectomy and fistula repair were performed. Adhesions were seen around the gallbladder, but there were almost no adhesions at the port sites from first operation. She had an uneventful postoperative course after the second operation and was discharged on postoperative day 5.

## 3. Discussion

The ideal surgical procedure for gallstone ileus remains controversial [6, 7]. Reported procedures include enterolithotomy alone, or with the addition of cholecystectomy and fistula repair. Treatment can be performed as a one-stage or two-stage procedure. One-stage surgery has been recommended to reduce the risk of recurrence of gallstone ileus postoperatively, to prevent cholecystitis, and because of the risk of gallbladder malignancy developing in the presence of a fistula [2]. However, one-stage surgery is associated with high mortality and morbidity. Recent studies have reported that enterolithotomy alone is associated with better outcomes [2, 3, 6], and laparoscopic surgery for gallstone ileus without cholecystectomy and fistula repair has resulted in fewer major complications and shorter hospital stay [2, 3]. Cholecystectomy and fistula repair are suggested in select patients [3, 6].

The transvaginal approach, especially for specimen extraction, is now increasingly performed to reduce the morbidity associated with abdominal wall destruction. This approach is considered safe, because it is established in gynecology [8, 9]. A meta-analysis reported that transvaginal hybrid NOTES has the advantages of less postoperative pain, low hernia risk, and faster recovery [10]. To our knowledge, ours is the first report to apply transvaginal hybrid NOTES to surgery for gallstone ileus. The diameter of our patient's gallstone was 3.5 mm, which would have required an abdominal incision greater than 3.5 mm for a conventional laparoscopic approach to extract the specimen and perform enterectomy. We elected to perform not only specimen extraction but also enterectomy via the vaginal route and obtained good results. A 12 mm port was created in the vagina, and enterectomy was performed using a linear stapler via the vaginal port. We accomplished laparoscopic surgery for gallstone ileus using only three 5 mm abdominal ports, relieving the patient's pain and obtaining good cosmetic outcomes.

Although transvaginal hybrid NOTES has certain advantages, there are also limitations. First, this procedure demands more time and skill than conventional laparoscopic surgery. Creating the vaginal route takes time, and totally laparoscopic enterectomy demands skills in intracorporeal anastomosis. Second, this procedure cannot be performed for every patient. If surgeons apply this procedure and encounter difficulty, a conventional approach should be adopted to secure patient safety. We believe that these limitations could be resolved with increased skill and improved endosurgical instruments. Further prospective studies are necessary to establish the indications for this procedure, but transvaginal hybrid NOTES may become an option to minimize invasiveness in other laparoscopic surgeries, in the future.
